# Development of pH-Responsive *N*-benzyl-*N*-*O*-succinyl Chitosan Micelles Loaded with a Curcumin Analog (Cyqualone) for Treatment of Colon Cancer

**DOI:** 10.3390/molecules28062693

**Published:** 2023-03-16

**Authors:** Sasikarn Sripetthong, Fredrick Nwude Eze, Warayuth Sajomsang, Chitchamai Ovatlarnporn

**Affiliations:** 1 Department of Pharmaceutical Chemistry, Faculty of Pharmaceutical Sciences, Prince of Songkla University, Hat Yai 90112, Songkhla, Thailand; ornlyy@hotmail.co.th (S.S.); fredrick.e@psu.ac.th (F.N.E.); 2 Drug Delivery System Excellence Center, Faculty of Pharmaceutical Sciences, Prince of Songkla University, Hat Yai 90112, Songkhla, Thailand; 3 Nanodelivery System Laboratory, National Nanotechnology Center, National Science and Technology Development Agency, Phathum Thani 12120, Thailand; warayuth@nanotec.or.th

**Keywords:** micelles, chitosan, 2,6-bis((3-methoxy-4-hydroxyphenyl) methylene) cyclohexanone, cyqualone, colon cancer

## Abstract

This work aimed at preparing nanomicelles from *N*-benzyl-*N*,*O*-succinyl chitosan (NBSCh) loaded with a curcumin analog, 2,6-bis((3-methoxy-4-hydroxyphenyl) methylene) cyclohexanone, a.k.a. cyqualone (CL), for antineoplastic colon cancer chemotherapy. The CL-loaded NBSCh micelles were spherical and less than 100 nm in size. The entrapment efficiency of CL in the micelles ranged from 13 to 39%. Drug release from pristine CL was less than 20% in PBS at pH 7.4, whereas the release from CL-NBSCh micelles was significantly higher. The release study of CL-NBSCh revealed that around 40% of CL content was released in simulated gastric fluid at pH 1.2; 79 and 85% in simulated intestinal fluids at pH 5.5 and 6.8, respectively; and 75% in simulated colonic fluid at pH 7.4. CL-NBSCh showed considerably high selective cytotoxicity towards mucosal epithelial human colon cancer (HT-29) cells and lower levels of toxicity towards mouse connective tissue fibroblasts (L929). CL-NBSCh was also more cytotoxic than the free CL. Furthermore, compared to free CL, CL-NBSCh micelles were found to be more efficient at arresting cell growth at the G2/M phase, and induced apoptosis earlier in HT-29 cells. Collectively, these results indicate the high prospective potential of CL-loaded NBSCh micelles as an oral therapeutic intervention for colon cancer.

## 1. Introduction

Cancer is a leading cause of morbidity worldwide, resulting in the death of an estimated 9.6 million persons in 2018 [1]. Colorectal cancers are one of the most prevalent types of cancer and second most common cause of cancer-related death in both males and females [2]. Cancer is conventionally treated by a number of approaches, including surgery, radiation and chemotherapy [3,4]. Intravenously injected chemotherapeutics are still the mainstay of cancer treatment [5]. However, systemic drug delivery following intravenous injection for treatment of colon cancer raises major problems of drug toxicity and side effects, including nausea and vomiting, diarrhea, decreased white blood cell count, anemia, fatigue, nerve damage, pain and skin reactions [6,7]. These complications severely diminish patients’ quality of life and present major obstacles to successful chemotherapy. Local delivery of cancer chemotherapeutics to tumor sites in the lung and breast following intravenous administration has the potential to reduce the administered dose and minimize adverse side effects by concentrating the drug at the site of action. Various intravenous drug delivery strategies have evolved to realize this potential, including antibody-directed enzyme prodrug therapy (ADEPT) [8,9] and targeting of drug-loaded nanoparticles to specific receptors on cancer cells [10,11] by incorporating ligands in the nanoparticle surface. However, phagocytic uptake of circulating nanomedicines, resulting in short blood residence times, has limited their application in clinically available therapeutics. The potential advantages of orally administered nanoparticles for delivery of drugs to the colon for treatment of inflammatory bowel disease and colorectal cancer are receiving increasing attention [12]. Nanoparticles less than 500 nm in size have been reported to traverse the mucosal layer and be taken up by solid tumors [13,14]. As a result, research on nanomedicines for applications in colon cancer therapy has increased significantly to include a variety of nanocarriers and nanoparticle in microcapsule systems.

Nanomicelles are self-assembled nano-sized structures, typically 10–200 nm in dimension, which may be produced from biodegradable and biocompatible amphiphilic block polymers and are therefore attractive as drug delivery systems. Micelles have previously been developed as intravenous nanocarriers for anticancer drugs to enhance ‘passive’ targeted delivery to tumor sites [15]. The micelles have been shown to easily pass through the ‘leaky’ vasculature associated with tumor tissues (extravasation) and elevate the drug concentration in the tumor. Biodegradation of the micelles avoids their accumulation in the cell, impairment of cellular processes and toxic side effects [7,16]. Chitosan has been widely investigated for the production of drug delivery systems in the form of carriers [17], due to favorable biocompatibility, biodegradability and low toxicity [18]. Curcumin-loaded *N*-benzyl-*N*,*O*-succinyl chitosan (NBSCh) micelles have been reported to exhibit intense cytotoxicity towards cervical cancer cells in vitro [19]. Curcumin is a hydrophobic polyphenol derived from the rhizome of the herb *Curcuma longa*. The compound has demonstrated a wide spectrum of biological and pharmacological activities, including antioxidant, anti-inflammatory, antimicrobial and anticancer [20,21]. However, curcumin’s therapeutic potential has been limited by poor solubility and instability under physiological conditions. 

**Figure 1:**
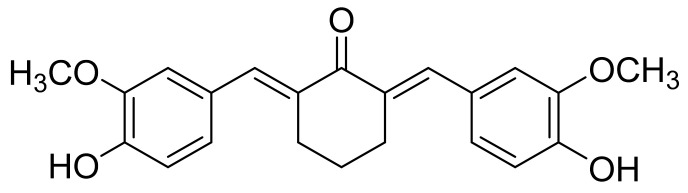
Structure of cyqualone (CL, 2,6-bis((3-methoxy-4-hydroxyphenyl) methylene) cyclohexanone).

Recently, new curcumin analogs were synthesized via structural modifications of the curcumin scaffold with enhanced physicochemical parameters. Some of the analogs were observed with enhanced bioavailability, due to enhanced transmembrane passage properties and delayed metabolism [22]. The curcumin analogue, 2,6-bis((3-methoxy-4-hydroxyphenyl) methylene) cyclohexanone (cyqualone (CL); Figure 1), was found to significantly decrease tumor cell proliferation and angiogenesis in adenocarcinoma of the rat prostate (PLS10)-bearing mice compared with curcumin [23,24]. CL has been reported to have cytotoxic activities against brain, breast, leukemia, pancreatic and prostate cancer cells, both in drug-sensitive and drug resistance cell lines, while exhibiting low toxicity in normal cells [23,24,25,26,27,28]. Due to limited bio-accessibility, bioavailability and stability of curcumin, a number of previous studies had focused on improving these crucial parameters using nanoparticle strategies [29,30,31,32,33,34,35]. Among these, nanomicelle preparation using chitosan derivatives is one of the most interesting approaches. For example, *N*-naphthyl-*N*,*O*-succinyl chitosan (NSCS) and *N*-octyl-*N*,*O*-succinyl chitosan (OSCS) nanomicelles containing curcumin were prepared [36,37] and found to be pH-sensitive and appropriate for colon-targeted drug delivery. The curcumin-loaded nanomicelles showed higher anti-colon cancer activity against HT-29 cells (IC_50_ = 6.18 ± 0.18 μg/mL) than plain curcumin (IC_50_ = 11.38 ± 3.07 μg/mL) [37]. 

In this study, we describe the development of a novel oral antineoplastic formulation based on *N*-benzyl-*N*,*O*-succinyl chitosan (NBSCh) micelle-encapsulated CL for efficacious colon cancer chemotherapy. The CL-loaded micelles were prepared by dialysis method and characterized in terms of size and morphology (TEM), chemical composition (NMR, FT-IR) and drug release behavior in simulated gastric and intestinal fluids. Furthermore, the anticancer activity of the micelles was thoroughly investigated using a human mucosal epithelial colon cancer cell line (HT-29). The findings from this work are expected to have positive implications in the effective treatment of colon cancer. 

## 2. Results and Discussion

### 2.1. Synthesis and Characterization of N-benzyl-N,O-succinyl Chitosan (NBSCh) 

NBSCh was successfully synthesized by reductive amination and succinoylation [19], resulting in a yellow powder with 63% product yield. The obtained *N*-benzyl chitosan (NBCh) and NBSCh were properly characterized using ^1^H-NMR (Figure S2) and FT-IR analysis (Figure S1), and the results are presented in Supporting Information.

Elemental analysis was used to calculate the degree of substitution of *N*-benzyl group and *N*,*O*-succinyl groups of NBCh. Data from the elemental analysis (Table 1) revealed that the structure of the chitosan starting material changed to NBCh according to substitution of benzyl groups for the N–atom of the amino group of chitosan. Analysis showed increasing %C and %H, but reducing %N. Moreover, the change in elemental compositions of NBSCh compared to that of NBCh confirmed the structural change after the reaction. NBSCh contained succinyl moiety on both N- and O atoms of the chitosan backbone, resulting in lower %C and %N compared to that of NBCh, whereas the higher %O of NBSCh relative to NBCh was due to the oxygen content of the substituting succinyl group in NBSCh. The elemental analysis data were used for calculation of the degree of substitution with succinyl radical (DSS) of NBSCh and resulting in 1.02 DSS.

### 2.2. Preparation and Characterization of Micelles

**Figure 2:**
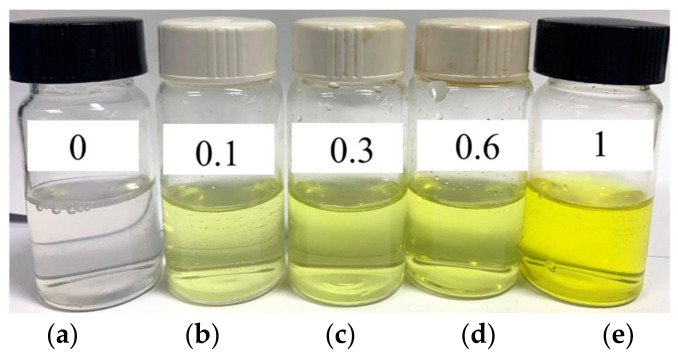
Pictures of blank NBSCh micellar solution (**a**) and NBSCh micellar solutions containing CL at different concentrations (**b**) 0.1, (**c**) 0.3, (**d**) 0.6, (**e**) 1.0 mg/mL.

The NBSCh is endowed with hydrophobic and hydrophilic segments on the chitosan backbone and therefore had the potential to form micelles through self-assembly. The dialysis method employed in this study yielded yellow-colored NBSCh micellar solutions (Figure 2). The mean size of the micelles in these solutions was 60.1 ± 1.20 nm and displayed a narrow size distribution (PDI value 0.096). The CMC of NBSCh was 0.014 mg/mL (Figure S3).

**Figure 3:**
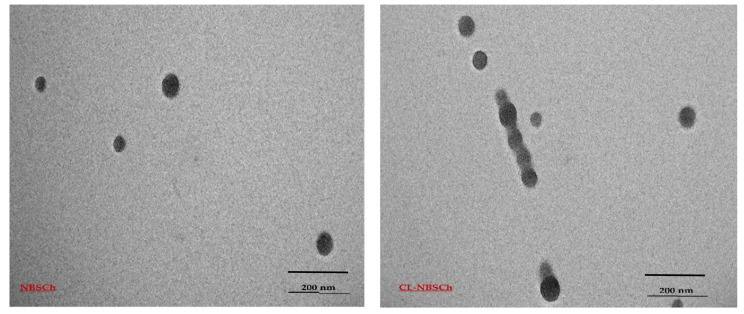
TEM micrographs of blank NBSCh and CL-loaded-NBSCh micelles.

Loading of some synthetic hydrophobic compounds with polymeric micelles have been observed, with a predominant increase in their pharmacological profile [38]. In the current study, the curcumin analog was successfully loaded into NBSCh micelles via hydrophobic–hydrophobic interactions between the cyqualone and hydrophobic core of the NBSCh micelles. TEM micrographs of CL-loaded NBSCh micelles revealed spherically shaped structures, with a consistent diameter of around 50 nm for both the blank and CL-loaded NBSCh micelles (Figure 3). Moreover, the size of CL-NBSCh was not markedly different from the blank samples. Size of the CL-micelles observed was 62.4 ± 0.9, zeta potential was −31.0 ± 0.6, and PDI was 0.236 ± 0.022. Nanoparticles with sizes less than 200 nm have been reported to be potentially useful carriers for anticancer drug delivery [39,40]. The CL-loaded NBSCh micelles were characterized by a negative surface charge, with zeta potential values around −30 mV. The negative charges of the succinyl moieties could prevent aggregation and improve stability of the drug-loaded micelles in aqueous media. Moreover, NBSCh possessed high numbers of carboxyl groups on its surface, such that NBSCh micelles could be mucoadhesive through interactions with mucus via hydrogen bonding, van der Waals interactions, polymer chain interpenetration and hydrophobic forces [19]. The effect of CL concentration on CL entrapment efficiency (EE) and loading capacity (LC) of NBSCh micelles were calculated according to Equations (1) and (2), respectively and the results are summarized in Table 2. The NBSCh concentration of the starting DMSO solution was kept constant at 1 mg/mL. The %EE remained constant (44.4–48.7%) with increasing CL concentration, indicating saturation of the available micelles. The loading capacity increased significantly from 4.25% to 32.75% with increasing CL concentration. Several factors in the drug were encapsulated in micelles: the hydrophobic interactions between CL and hydrophobic moieties of NBSCh, and the miscibility between NBSCh and CL. If CL interacted with the hydrophobic polymer chain more than with solvent, high-incorporation efficiency would be observed. However, if CL interacted with the hydrophobic polymer chain less than the solvent, CL would be precipitated [37,41].

(1) $$\%\mathrm{EE}=\left(\frac{wt\;of\;\mathrm{CL}\;encapsulated\;in\;micelles}{wt\;of\;\mathrm{CL}\;used\;for\;micelles\;preparation}\right)\times 100$$

(2) $$\%\mathrm{LC}=\left(\frac{wt\;of\;\mathrm{CL}\;encapsulated\;in\;micelles}{Total\;wt\;of\;the\;obtained\;dried\;micelle}\right)\times 100$$

CL-loaded NBSCh micelles were prepared by mixing solutions of CL stock solution (100, 300, 600 and 1000 µL of 10 mg/mL in DMSO) with NBSCh in DMSO (1 mg/mL) according to the information in Table 2, followed by dialysis against distilled water. The NBSCh concentration of the starting DMSO solution remained constant at 1 mg/mL.

### 2.3. In Vitro Release of CL from NBSCh Micelles

**Figure 4:**
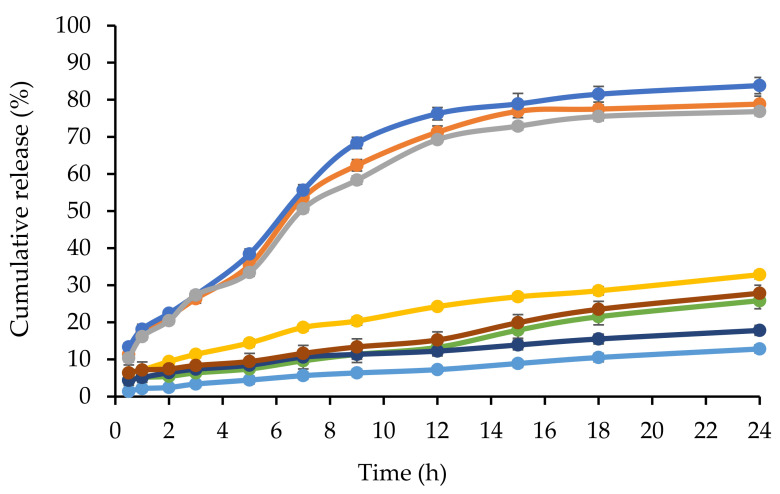
Dissolution profiles at 37 °C during 24 h of CL in simulated gastric fluid (SGF pH = 1.2, ●), simulated extracellular tumor cells fluid (ETC, pH 5.5, ●), simulated intestinal fluids (SIF, pH = 6.8, ●) and simulated colon fluid (SCF, pH = 7.4, ●) and CL from CL-loaded NBSCh micelles in SGF pH 1.2 (●), ETC, pH 5.5 (●), pH 6.8 (●) and SCF, pH = 7.4 (●).

**Figure 5:**
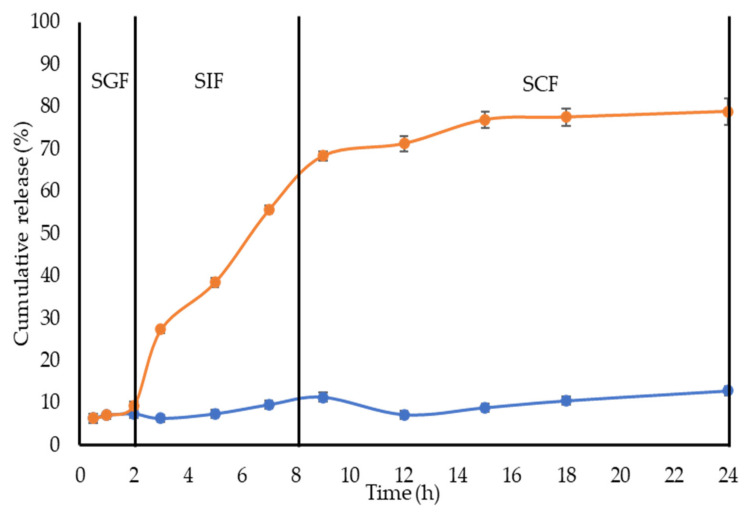
Gastrointestinal-simulated cumulative release profiles of CL (●) and CL from CL-loaded NBSCh micelles (●) in SGF pH 1.2 (0–2 h), followed by SIF pH 6.8 (2–8 h) and finally in SCF pH 7.4 (8–24 h).

**Figure 6:**
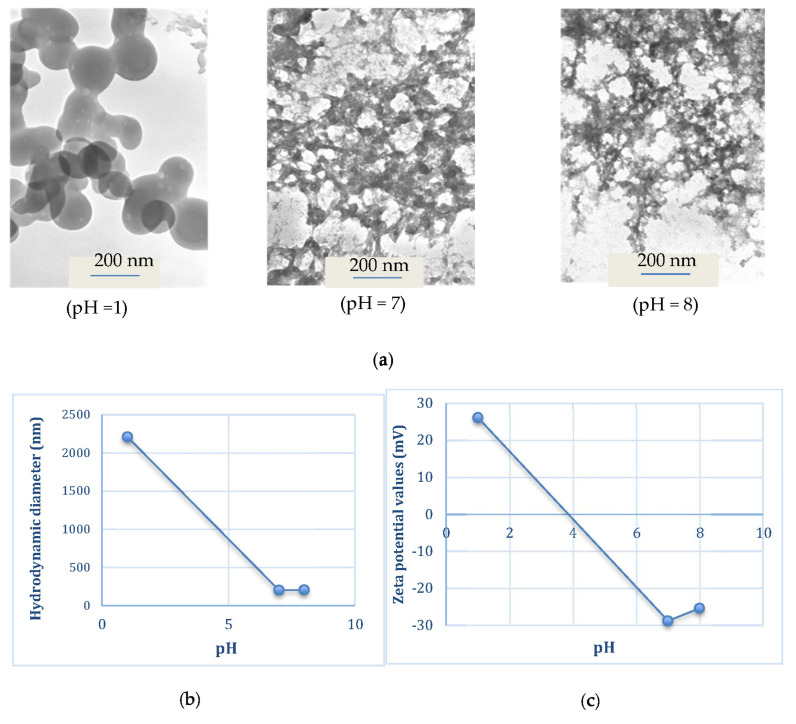
TEM micrographs (**a**), particle sizes (**b**) and zeta potentials (**c**) of 1 mg/mL blank NBSCh micelle in different pH mediums.

The release profiles of CL in gastric fluid (SGF), simulated extracellular tumor cells fluid (ETC, pH 5.5), simulated intestinal fluid (SIF, pH 6.8) and simulated colon fluid (SCF, pH 7.4) are shown in Figure 4 and Figure 5. Free CL dissolved slowly in each medium, reaching a maximum of 28% (pH = 1.2), 20% (pH = 5.5), 26% (pH = 6.8) and 16% (pH = 7.4) after 18 h. The release profile of CL from CL-loaded NBSCh micelles in SGF (pH = 1.2) showed a similar pattern to that obtained for the dissolution of CL in SGF. However, the amount of CL in ETC (pH 5.5), SIF (pH 6.8), and SCF (pH 7.4) releasing from CL-loaded micelles was significantly higher than that from free CL. The maximum release of CL from CL-NBSCh micelles in pH 5.5, pH 6.8 and pH 7.4 media was found to be 79, 85 and 75%, respectively (Figure 4 and Figure 5). This behavior may be influenced by the pKa_1_ of 4.21 [42] of succinic moieties in the micelles. At pH 5.5, 6.8 and 7.4, the succinic groups would be ionized, possibly resulting in a loose micelle structure, which facilitated the release of the entrapped CL [19,37]. The release behavior of CL was found to be dependent on the pH of the release medium. TEM morphologies, particle sizes and zeta potentials findings confirmed that morphologies, sizes, and zeta potentials change of the micelles varied with pH. In acidic medium, the succinic acid were in unionized form, whereas, at pH lower than 4.1, the pKa_1_ of succinic acid, the micelle aggregated like a cluster, resulting in larger-sized nanomicelles, leading to larger hydrodynamic diameter with a positive charge (Figure 6b,c). The TEM image of the micelles in a low pH medium revealed a spherical shape, suggesting that the micelles retained their integrity and modulated the release of the drug under strong acidic condition at a slow pace (Figure 6a). As such, the prepared NBSCh micellar system could be useful for improving the bioavailability of the drug. 

Unlike in the stomach, where the micelles could retain their spherical morphology, on reaching the simulated small intestine and colon with pH values in the range of 6.8–7.4, the micelles are dissociated, facilitating the release of their drug load. The pH values of the intracellular compartments of tumors are within 5.0–6.5, and the tumor extracellular environment are in the range of pH 6.5–7.2 [43]. Therefore, the NBSCh micelles can be adapted towards pH-triggered accelerated drug release at intracellular sites, such as the acidic tumor tissues or tumor cells. Interestingly, the solubility improvement of the drug could enhance its bioavailability. Xu et al. (2015) reported that quercetin loaded MPEG–PCL micelles could improve drug release, and an in vivo study showed that quercetin loaded MPEG–PCL micelles could enhance the T_1/2_ and C_max_ of quercetin and could better improve the colon cancer cytotoxicity than pure quercetin [44].

### 2.4. Effect of Temperature on the Stability of CL-Loaded NBSCh Micelle Powders 

**Figure 7:**
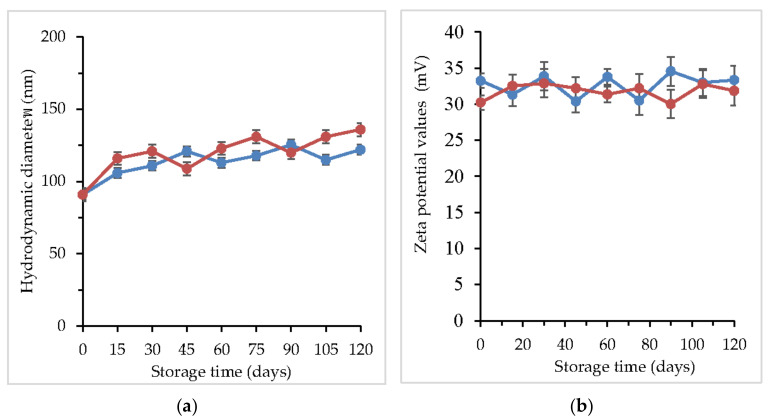
Sizes of CL-loaded NBSCh micelles following redispersion of powders in distilled water (**a**). Zeta-potential values of the CL-loaded NBSCh micelles freeze-dried powders during storage (**b**) stored at 4 °C (●) and 30 °C (●) for 120 days.

The storage stability of CL-loaded NBSCh micelle powders was evaluated by determining the sizes, zeta potentials of the micelle, and the content (%) of CL remaining at the end of the storage period (Figure 7). Redispersion of dried powders in distilled water resulted in an increase in micelle size to around 130 nm after 15 days’ storage, compared to 100 nm of non-freeze-dried preparations. Storage for 120 days at 30 °C resulted in a slight increase in size (135 nm) compared with storage at 4 °C (120 nm), indicating a fairly good storage stability. All samples showed only a slight increase in zeta potential values during storage, providing a further indication of their stability (Figure 7a). The storage stability of the CL-loaded micelle powder samples at 4 and 30 °C was further investigated by measuring the amount of CL that remained at various storage times (Figure 7b). More than 90% of the initial content of CL was found remaining after 120 days’ storage, demonstrating superb stability of the freeze-dried micelle powders containing glycine as a cryoprotectant.

### 2.5. CL-Loaded NBSCh Micelles Were Selective and Cytotoxic to HT-29 Colon Cancer Cells

**Figure 8:**
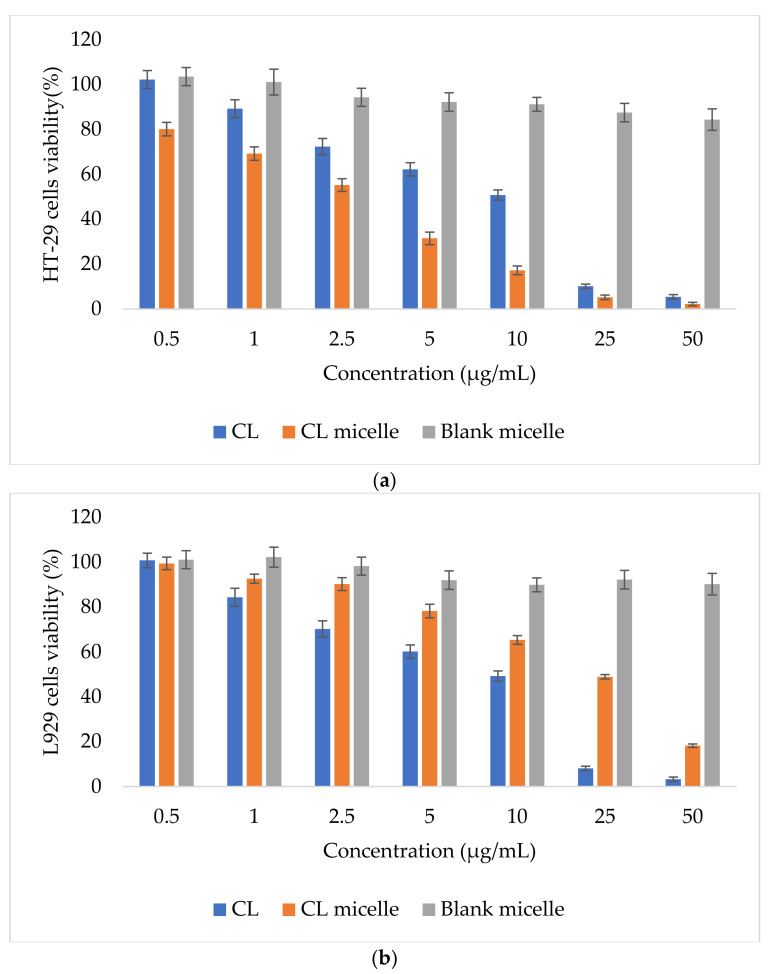
HT-29 (**a**) and L929 (**b**) cells’ viability (%) of the CL (■), CL micelle (■) and blank micelle (■) by MTT assay.

Cyqualone has been previously shown to reverse P-gp-mediated multidrug resistance (MDR) at a low dose (2.5 µM), which is potentially advantageous for avoiding side effects in chemotherapy [21]. The compound was also reported to exhibit higher anti-invasion properties against castration-resistant prostate cancer cells than curcumin [45] and inhibited both MMP-2- and MMP-9-defined MMP activities [46]. In the present study, blank micelles showed minimal toxicity towards both normal (L929) mouse fibroblasts and human colon cancer (HT-29) cells. Increasing micelle concentrations from 1–200 µg/mL resulted in a gradual reduction in viability of both cell types but remained over 80%. Figure 8 demonstrates HT-29 (a) and L929 (b) cells’ viability (%) of the CL, CL micelle and blank micelle by MTT assay. The cytotoxic effect of CL and CL-loaded NBSCh micelles against human colon mucosal epithelial cancer cells (HT-29) and mouse connective tissue fibroblasts (L929) in terms of IC_50_ values is summarized in Table 3. Free CL demonstrated similar toxicity towards both human colon cancer cells and normal mouse fibroblasts (IC_50_ approximately 10 µg/mL). CL-loaded micelles, on the other hand, exhibited significantly higher toxicity against the cancer cell line (IC_50_ = 3.4 µg/mL) compared to free CL by a factor of three. This behavior suggests that the micelle carrier is taken up efficiently by colon cancer cells, thus enhancing the transport of CL. Kansom et al. (2018) prepared andrographolide (or 3A.1)-loaded naphthyl-grafted succinyl chitosan (NSC), octyl-grafted succinyl chitosan (OSC) and benzyl-grafted succinyl chitosan (BSC) nanopolymeric micelles. Similarly, to our results, they found that 3A.1-loaded nanopolymeric micelles showed significantly lower IC_50_ against HT-29 colon cancer than the pure drug [47]. Importantly, in the present study, CL-loaded micelles were found to demonstrate substantially high levels of selectivity, as indicated by the IC_50_ value of 24.3 µg/mL obtained when tested against mouse fibroblasts compared to 3.4 µg/mL against colon cancer cells. In addition, the toxicity of CL-loaded micelles to normal cells was almost four times lower than that of CL, suggesting a shielding effect by the micelle core. This could be due to a nanomicelle with a size about 60 nm being potentially better at entering cancer cells via the pore of the epithelial cell of the cancer cell and better preventing the EPR effect than a normal cell; nanomicelles could also prevent the elimination of pure compounds via the EPR effect. 

### 2.6. Cellular Uptake of CL-Loaded NBSCh Micelles

**Figure 9:**
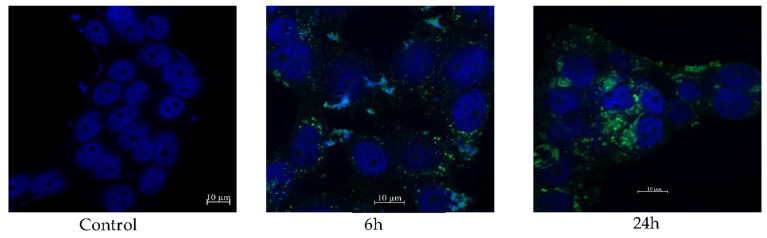
CLSM analysis of NBSCh micelles uptake by human colon cancer cells (HT-29) 6 h and 24 h after exposure. Blue fluorescence represents the cell nuclei stained by Hoechst 33,342 stain. Green fluorescence represents the micelles labelled by FITC.

The cellular uptake of NBSCh micelles by human colon mucosal epithelial cancer cells (HT-29) was monitored following exposure for 6 and 24 h using CLSM (Figure 9). Blue fluorescence was used to identify the cell nucleus stained by Hoechst 33,342 dye, while green fluorescence was used to identify the micelles labelled by FITC. Greater cellular uptake was apparent after 24 h compared with 6 h, and NBSCh micelles had moved very close to the nucleus at 24 h. The CLSM study indicated that NBSCh micelles were able to penetrate the cell membrane, possibly by caveolae-mediated endocytosis, and enter the cytoplasm [15,16]. Micelles’ translocation may be facilitated by their anionic surface charge, which favors interaction with cationic lipid domains in the cell membrane.

### 2.7. CL-Loaded NBSCh Micelles Promoted Early Apoptosis in HT-29 Cancer Cells 

**Figure 10:**
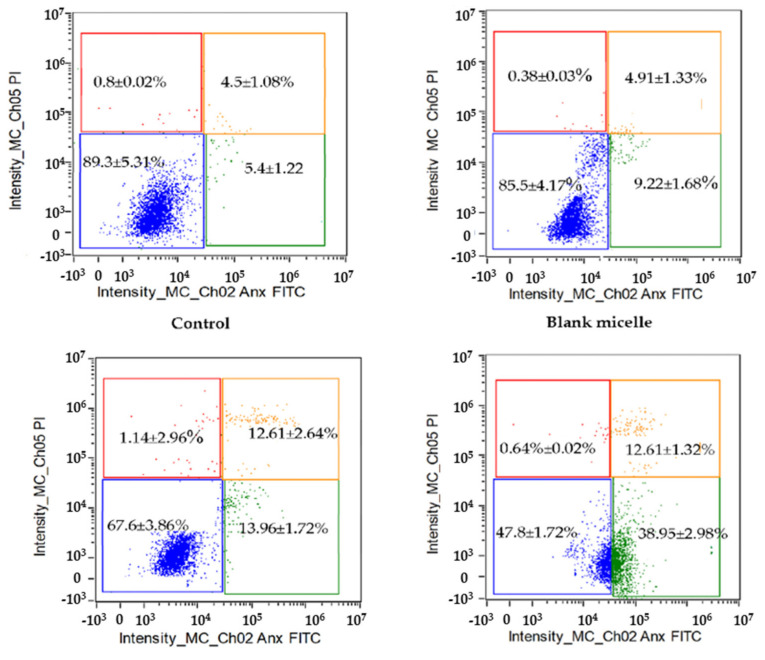
The effect of curcumin analog (CL) and CL-loaded NBSCh micelles on induced apoptosis of HT-29 human colon cancer cells. Lower left quadrant represents viable cells that do not bind to AnnexinV-FitC or PI; lower right quadrant represent early apoptotic cells binding to only AnnexinV-FitC; top right quadrant represent necrotic or late apoptotic cells that were both AnnexinV-FitC- and PI-positive; and top left quadrant represent necrotic cells binding to only PI.

During apoptosis, phagocytes receive signals via phosphatidylserine (PS) in the extracellular membrane—for example, to initiate cell clearance. Annexin V binds explicitly to the PS of apoptotic cells in early apoptosis, whereas propidium iodide (PI) can stain the nucleus of late apoptosis (necrotic) cells due to loss of membrane integrity. HT-29 cells were exposed to free CL and CL-loaded NBSCh micelles for 24 h at the CL IC_50_ value of 10.6 µg/mL for free CL and 3.4 µg/mL for CL in micelles. Double staining using Annexin V-FITC and PI, followed by flow cytometry, was performed to distinguish between early apoptosis and necrosis. Viable cells do not bind to AnnexinV-FITC or PI. Flow cytometry analysis demonstrated that HT-29 colon cancer cells retained more than 85% cell viability following exposure to culture media (control) or blank micelles. Early apoptotic cells (AnnexinV- FITC bound and negative for PI (lower right quadrant)) were found to represent 14.0 ± 1.7% of the population following exposure to CL, while CL-loaded NBSCh micelles induced a significant 3-fold increase in early apoptosis (39.0 ± 3.0% of the total population) compared to free CL. This behavior is consistent with earlier observations pointing towards the efficient uptake of NBSCh micelles by HT-29 cancer cells (Section 2.6), and indicated the potential of CL-loaded micelles for application in cancer chemotherapy. Necrotic or late apoptotic cells are expected to be positive for both AnnexinV-FITC and PI (Figure 10, upper right quadrant). This accounted for approximately 12.6% of the cell population (Figure 10, upper right quadrant). These observations indicated that, after 24 h exposure to CL or CL-loaded micelles, most of the HT-29 cells had not entered the late apoptosis phase.

### 2.8. Cell Cycle for Antiproliferative Effect of CL and CL-Loaded Micelles on HT-29 Human Colon Cancer Cells 

**Figure 11:**
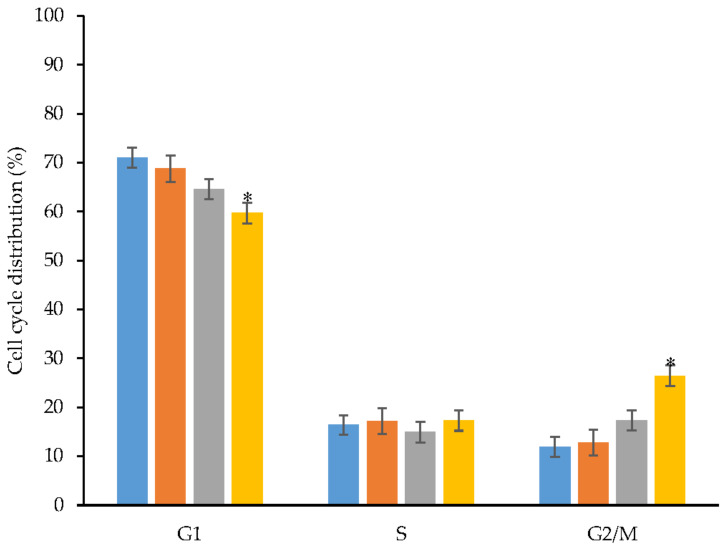
Cell cycle distribution of HT-29 human colon cancer cells exposed for 24 h to culture medium (control) (■), blank NBSCh micelles (■), free curcumin analog (■) and CL-loaded NBSCh micelles (■). * < 0.05 (*n* = 3).

The cell cycle is a process where a cell grows and divides to create a copy of itself. The cell cycle involves four main phases: G1, S, G2 and M phases. G1 is the starting phase, when the cell is ready to get into DNA replication before entry into the S phase. The S phase is where DNA is replicated. Once DNA has been replicated, the cell enters G2 phase, which prepares the cell for division. Finally, the cell is divided during M phase. In this study, flow cytometry was employed to understand the antiproliferative effects of free CL, blank NBSCh micelles and CL-loaded NBSCh micelles on the cell cycle of HT-29 human colon cancer cells. Formulations containing CL were added to the cell cultures to provide an IC_50_ value of CL 10.6 µg/mL (pure CL) and 3.4 µg/mL (CL in micelles). The DNA content of the cells following 24 h exposure was analyzed by flow cytometry after labelling with propidium iodide (PI) and converted to % cell number in each cell cycle (Figure 11). 

The blank NBSCh micelles exhibited no significant effect on the cell cycle compared with untreated controls. CL-loaded micelles caused a significant reduction in cell number in the G1 phase (approximately 60% of the population, compared with 70% for control samples), but no effect was observed in the S phase. Approximately 15% of the total cell population existed in the S phase of growth. The percentage of cells in the G2/M phase was significantly increased from around 17.32 ± 2.64% to 26.42 ± 2.76% following exposure to CL-loaded NBSCh micelles for 24 h, compared with free CL, indicating an improvement in the drug-loaded micelle ability to induce cell cycle arrest and exert a 1.53-fold increase in the inhibition of cancer cell division (Figure 11) [40,48]. This observation correlated well with the efficient uptake of CL-loaded NBSCh micelles (Section 2.6) and the significant 3-fold increase in early apoptosis (39.0 ± 3.0 % of the total population) compared with free CL (Section 2.7). Overall, these findings demonstrated that CL-loaded micelles hold high promise for applications in colon cancer chemotherapy. 

## 3. Materials and Methods

### 3.1. Chemicals 

The curcumin analogue, 2,6-bis((3-methoxy-4-hydroxyphenyl) methylene) cyclohexanone (cyqualone, CL, >99% purity) was previously synthesized, purified and well characterized in our lab. Chitosan with molecular weight of 30,000 g/mol and ≥80% degree of deacetylation was obtained from Bannawach Bio-Line Co. Ltd., Bangkok, Thailand. Benzaladehyde, analytical grade, was purchased from Panreac., Barcelona, Spain. Glacial acetic acid, methanol, dimethylformamide and sodium hydroxide were purchased from Labscan Asia, Bangkok Thailand. 4-Nitrobenzoyl chloride was obtained from Fluka, Munich, Germany. Succinic anhydride and sodium cyanoborohydride were obtained from Sigma-Aldrich, Munich, Germany. 

### 3.2. Reagents and Cell Lines for Cytotoxicity Studies 

Mucosal epithelial human colon cancer cell line (HT-29) and mouse connective tissue fibroblasts (L929) were purchased from ATCC^®^, Manassas, VA, USA. Dulbecco’s Modified Eagle’s Medium (DMEM), fetal bovine serum (FBS) and 0.25% trypsin–EDTA (1X) were purchased from Gibco (Invitrogen, Carlsbad, CA, USA). Phosphate buffered saline pH 7.4 (PBS) was purchased from Sigma–Aldrich, Munich, Germany. 3-[4,5-Dimethylthiazol-2-yl]-2,5-diphenyltetrazolium bromide (for MTT assay of cell viability) and Hoechst 33,342 were purchased from Invitrogen^TM^ Molecular Probes^®^, Eugene, OR, USA. Dimethyl sulfoxide (DMSO) was of analytical grade and purchased from Labscan, Bangkok, Thailand. Guava Cell Cycle Reagents for Flow Cytometry was purchased from Merck, Darmstadt, Germany and FITC Annexin V Apoptosis Detection Kit I was obtained from BD Pharmingen^TM^, Franklin Lakes, NJ, USA.

### 3.3. Synthesis of N-benzyl-N,O-succinyl Chitosan (NBSCh)

**Figure 12:**
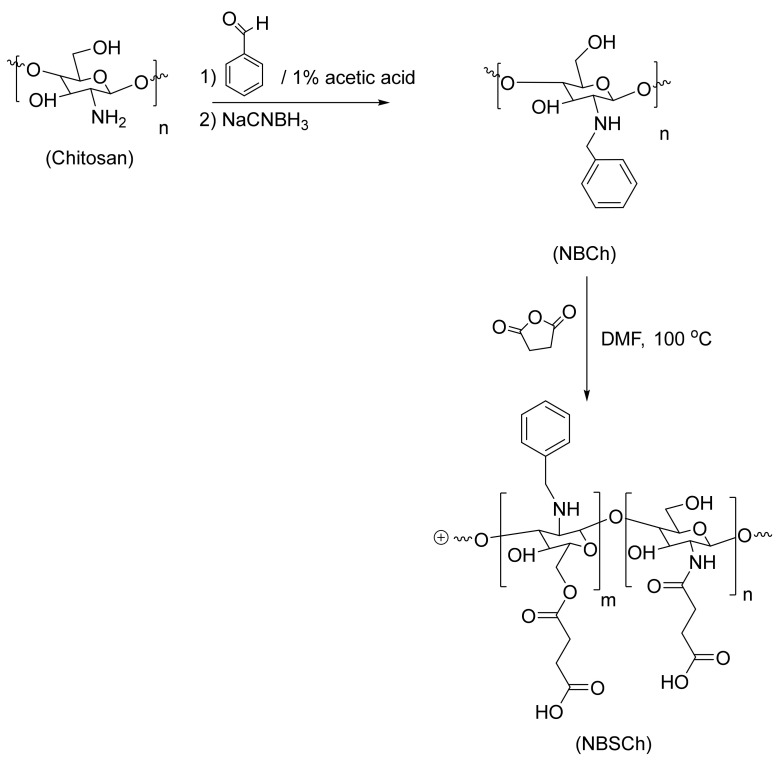
Synthesis scheme for *N*-benzyl-*N*,*O*-succinyl chitosan (NBSCh).

*N*-benzyl-*N*,*O*-succinyl chitosan (NBSCh) was synthesized by reductive amination and succinoylation in two stages, according to a previous report [19] (Figure 12). Chitosan 10 g (equivalent to 0.35 mol of glucosamine) was dissolved in 500 mL 1% acetic acid at room temperature. A solution of benzaldehyde in ethanol (0.06 g/mL, 300 mL) was added and the mixture was stirred continuously at room temperature for 24 h. NaOH (15%, 1 mL) was then added to adjust the pH of the mixture to 5. Subsequently, sodium cyanoborohydride (1.89 g, 0.03 mol) was added to the mixture with further stirring at room temperature for 24 h. The resulting solution was subjected to dialysis (cellulose membrane MW cut off 12,000–14,000, Membrane Filtration Products, Seguin, TX, USA) against distilled water for 3 days. The dialyzed solution was lyophilized (freeze dryer, DELTA 2-24 LSC, Darmstadt, Germany), resulting in a white powder of *N*-benzyl chitosan (NBCh). NBCh was characterized using FT-IR on Perkin-Elmer FT-IR model 1600 spectrometer (Llantrisant, UK) and NMR on FT-NMR spectrometer (500 MHz, Unity Inova, Varian, Darmstadt, Germany). 

*N*-benzyl-*N*,*O*-succinyl chitosan (NBSCh) was then synthesized by succinoylation as described. Briefly, *N*-benzyl chitosan NBCh (5 g) was dispersed in 450 mL dimethylformamide (DMF) 450 mL, and succinic anhydride (15 g, 0.30 mol) was added. The reaction mixture was stirred under nitrogen purge for 24 h, resulting in a clear yellow solution. Excess of succinic anhydride and DMF were eliminated by dialysis against distilled water for 3 days. The final solution in the dialysis bag was freeze-dried to give a yellow powder prior to characterization by FT-IR and ^1^H-NMR. 

### 3.4. Preparation of Blank NBSCh Micelles and CL-Loaded NBSCh Micelles

Blank NBSCh micelles were prepared using a dialysis method. A solution of 10 mg of NBSCh in 10 mL DMSO was prepared at room temperature and subjected to dialysis against distilled water (cellulose membrane, MW. cut off = 3500) for 18 h. The solution in the dialysis tubing was filtered using a nylon-membrane syringe filter (pore size = 0.22 µm, Vertical Chromatography, Bangkok, Thailand). The CL-loaded NBSCh micelles were prepared by using the same procedure as the blank as follows: NBSCh powder 10 mg was dissolved in in DMSO (10 mL) at room temperature. Different concentrations of CL solutions in DMSO (100, 300, 600 and 1000 μL of 10 mg/mL prepared as solutions, having final concentrations of 0.1, 0.3 0.6 and 1 mg/mL, were added to each tube of the NBSCh solution while stirring at room temperature for 1 h to provide clear yellow solutions. The resulting solutions were then dialyzed against distilled water for 24 h (cellulose membrane, MW cut off = 3500), and the final micellar solution was filtered through 0.22 µm PVDF filter (Vertical, Bangkok, Thailand). Finally, glycine was added to the filtrates to obtain a final concentration of 1% *w*/*v* before freeze-drying (freeze drier, DELTA 2-24 LSC, Darmstadt, Germany) to produce light yellow powder.

### 3.5. Determination of Critical Micelle Concentration (CMC)

The Critical Micelle Concentration (CMC) of NBSCh micelles was determined by means of a fluorescence spectroscopic technique. NBSCh solutions in distilled water (4 mL) at various concentrations (0.002–1 mg/mL) were placed in separate tubes, and 0.1 mM pyrene in acetone (10 µL) was added to each sample. The mixtures were sonicated at room temperature for 15 min, followed by heating at 50 °C for 2 h, before being kept overnight in the dark at room temperature to reach equilibrium. The fluorescence intensity of the samples was measured using a fluorescence spectrophotometer (Cary Eclipse, Perkin Elmer Ltd., Wokingham, UK) at an excitation wavelength of 335 nm. The emission intensities were recorded at 373 and 382 nm to investigate the shift in NBSCh hydrophobic microdomains, by monitoring the change in intensity ratio (I_1_/I_3_) at 373 nm (I_1_) and 382 nm (I_3_). The CMC (mg/mL) was calculated after fitting the semi-log plot of the intensity ratio I_1_/I_3_ vs. concentrations of NBSCh. 

### 3.6. Physicochemical Characterization of CL-Loaded NBSCh Micelles

#### 3.6.1. Size and Zeta Potential Measurements

The micelle size, size distribution, and zeta potential of CL-loaded NBSCh micelles were measured by dynamic light scattering using a Zetasizer Nano ZS (Zetasizer Nano ZS, Malvern, UK). Samples were freshly prepared in distilled water and filtered through a 0.22 µm filter prior to analysis.

#### 3.6.2. Micellar Morphology Determination

The shape and surface appearance of the CL-loaded NBSCh micelles were observed by transmission electron microscopy (TEM, JEM-2010, JEOL, Tokyo, Japan). The micellar solutions in distilled water were dropped onto copper grids (200 mesh) and stained with uranyl acetate solution (1%, *w*/*v*) for subsequent TEM examination.

### 3.7. The Entrapment Efficiency (EE) and Loading Capacity (LC) of CL in NBSCh Micelles

The entrapment efficiency and loading capacity of CL-loaded NBSCh micelles was determined by using HPLC. The test sample was prepared by mixing CL-loaded NBSCh micellar solution with DMSO to give a clear yellow solution. The CL content of the test samples was estimated by a HPLC system consisting of a Chromaster 5110 pump and a Chromaster 5430 Diode Array Detector. The output signal was monitored and processed using a Chromaster 60 MPa System. A Phenomenex^®^ C18 250 mm × 4.6 mm, 5 µm column was used with a mobile phase containing acetonitrile and 0.01 mM trifluoroacetic acid at the ratio of 30:70 (*v*/*v*). The flow rate of the mobile phase was 1.5 mL/min. The detection wavelength was set at 385 nm. The entrapment efficiency (%) and loading capacity (%) of CL in the micelles were quantified by comparison of elution peak areas obtained for CL-micellar samples with the chromatograms of standard solutions of CL in acetonitrile. 

### 3.8. In Vitro Release of CL from NBSCh Micelles 

In vitro release of CL from NBSCh micelles was evaluated using a dialysis method in simulated gastric fluid [SGF, (HCl, pH 1.2)], simulated intestinal fluid [SIF, (PBS, pH 6.8)], extracellular tumor cells [ETC (PBS, pH 5.5)] and simulated colonic fluid [SCF, (PBS, pH 7.4)]. Solutions of CL-loaded NBSCh micelles were prepared in each release medium to obtain a CL concentration of about 50 µg/mL, and 5 mL of each solution was transferred to a dialysis tube (MW cut off 3.5 kDa) and immersed into 50 mL of each respective release medium. The release study was performed at 37 °C in an incubator (ES80 shaker, Grant Instruments, Cambridgeshire, UK) shaking at 120 rpm. At intervals of 0.5, 1, 2, 3, 5, 7, 9, 12, 15, 18 and 24 h, 1 mL of the released medium outside the dialysis tube was collected and replaced with 1 mL of a fresh medium at the same temperature. The amount of CL released from the NBSCh micelles was quantified by HPLC, and the release behavior was presented as a cumulative release (%) versus time. All drug release tests were performed in triplicate.

High-performance liquid chromatography (HPLC) was carried out using a Hitachi system based on a Chromaster 5110 pump and a Chromaster 5430 Diode Array Detector. The output signal was monitored and processed using a Chromaster 60 MPa. 

### 3.9. Determination of Stability of Freeze-Dried CL-Loaded NBSCh Micelle

The storage stability of CL-loaded NBSCh micelle powders was investigated by storing solid samples in well-closed vials at 4 °C and 30 °C for 16 weeks. After the indicated storage time, the samples were taken and evaluated for their sizes and zeta potentials. The CL content of each sample was determined by mean of HPLC. Samples were prepared in the same manner as %EE and %DL investigation.

### 3.10. Anticancer Activity of CL-Loaded NBSCh Micelle

#### 3.10.1. Determination of In Vitro Cytotoxicity of CL-Loaded NBSCh Micelles

The mucosal epithelial human colon cancer cell line (HT-29) and mouse connective tissue fibroblasts (L929) were maintained in Dulbecco’s Modified Eagle Medium (DMEM) containing 10% (*v*/*v*) fetal bovine serum [FBS]. Sub-confluent monolayers were trypsinized and seeded at a density of 2 × 10^4^ cells/well in 96-well plates and grown in 10% FBS DMEM at 37 °C for 24 h in a 5% CO_2_ atmosphere. The cytotoxicity activity of the test samples was determined using the 3-[4,5-dimethylthiazol-2-yl]-2,5-diphenyl tetrazolium bromide (MTT)-based colorimetric assay, which quantifies the number of viable cells after 24 h. The exponentially growing cells were washed twice with PBS (pH = 7.4) and incubated for 24 h with 100 µL of fresh medium (control) or medium containing CL or CL-loaded NBSCh micelles. Cell survival was assessed by adding MTT solution (50 µL of 5 mg/mL MTT in PBS) and incubated for 4 h. DMSO (100 µL) was then added to dissolve the formazan crystals formed. Absorbance (Ab) of the samples in the 96-well plate was measured by a microplate reader (SPECTRO star Nano, Beckman Coulter, Raleigh, NC, USA) at 570 nm. Percentage cell viability was calculated using the following equation, Equation (3):

(3) $$Cytotoxicity\;\left(\%\right)=\frac{\left({\mathrm{Ab}}_{570}control-{\mathrm{Ab}}_{570}sample\;\right)\times 100}{{\mathrm{Ab}}_{570}control}$$

#### 3.10.2. Determination of Cellular Uptake of NBSCh Micelles

Cellular uptake of NBSCh micelles was performed following conjugation with fluorescein isothiocyanate (FITC). Briefly, NBSCh (3 mmol) was reacted with 0.06 mmol *N*-hydroxysuccinimide (NHS) and 0.036 mmol *N*,*N*′-dicyclohexylcarbodiimide (DCC) in 20 mL of DMSO at room temperature for 12 h. Ethylenediamine (0.03 mmol) was added to the mixture and underwent continuous stirring for 2 h, after which FITC solution in DMSO (12 mg/5 mL) was added, with continuous stirring for 24 h to complete the reaction. The resulting mixture was dialyzed against distilled water to remove free FITC to obtain FITC-labelled NBSCh micelles. Cellular uptake of FITC-labelled NBSCh micelles was investigated using a confocal laser scanning microscopy (Zeiss LSM 800, Zeiss, Germany). HT-29 cells at a concentration of 3 × 10^5^ cells/well (1 mL) were seeded onto sterilized glass coverslips and incubated overnight in a 6-well plate for 24 h. Thereafter, the medium was removed and washed twice with PBS (pH 7.4). FITC-labelled micelles in 10% FBS DMEM medium (250 µg/mL, IC_50_ value of blank NBSCh micelles) was added and incubated at 37 °C under 5% CO_2_ for 6 and 24 h. The cells were then washed twice with PBS (pH 7.4), fixed with cold 70% ethanol for 10 min and washed twice with PBS (pH 7.4). Hoechst 33,342 dye (2’-[4-ethoxyphenyl]-5-[4-methyl-1-piperazinyl]-2,5’-bi-*1H*-benzimidazole trihydrochloride trihydrate) was used to stain the cellular nuclei. The Hoechst 33,342 solution (10 mg/mL) was diluted in sterile water at a ratio of 1:1000 *v*/*v* and 2 mL of this solution was added to the fixed cells in each well. After 30 min of incubation at 37 °C under 5% CO_2_, the solution above the cells was aspirated and the cells were washed twice with PBS (pH 7.4). The fixed cells attached to the coverslips were examined using a confocal laser scanning microscopy (Zeiss LSM 800, Zeiss, Jena, Germany). 

#### 3.10.3. Cell Apoptosis Study

Cell apoptosis was investigated by using annexin V-FITC/propidium iodide (PI) double staining to detect both apoptotic and necrotic cells. HT-29 cells were maintained in 10% FBS DMEM medium to attain a density of 80% confluence. Then, sub-cultured monolayers were trypsinized and cells were seeded into a 6-well culture plate (1 × 10^6^ cells/well). After 24 h, cells were washed twice with PBS (pH = 7.4) and incubated with 1 mL of blank micelles or CL-loaded NBSCh micelles containing CL at the IC_50_ value against HT29 cells. The cells were incubated at 37 °C for 24 h in a 5% CO_2_ atmosphere. Following incubation, the cells were dispersed using trypsin–EDTA solution, centrifuged at 500× *g* for 5 min and the cell pellets were re-suspended in PBS (pH 7.4). The cells were then stained with annexin V–FITC (5 µL) and PI (3 µL) by incubating for 15 min in the dark at room temperature. Finally, 100 µL of annexin V binding buffer was added. Cells were analyzed using a flow cytometer (Amis Image X-MKII system, Merck, Germany), with green fluorescence (FITC) detected at an excitation wavelength of 535 nm and red (PI) fluorescence detected at 550 nm wavelength. 

#### 3.10.4. Cell Cycle Analysis for Antiproliferative Effect of CL and CL-Loaded Micelles

Flow cytometry was carried out to investigate the antiproliferative effect of CL and CL-loaded NBSCh micelles on the cell cycle. HT-29 cells (1 × 10^6^ cells/well) were seeded in 6-well culture plates and incubated for 24 h in the presence or absence of CL or CL-loaded NBSCh micelles at the IC_50_ value of CL 10.6 µg/mL (pure CL) and 3.4 µg/mL(CL in micelles). Following incubation, the cells were trypsinized, washed twice with PBS and centrifuged at 500× *g* for 5 min. The collected cells were fixed using 70% ethanol at 4 °C overnight and washed twice with PBS. The cell pellets were re-suspended with 200 µL of PI and incubated at 30 °C in the dark for 30 min. Finally, the cells were analyzed using Flow cytometer (Amis Image X-MKII system, Merck, Germany).

### 3.11. Statistical Analysis

Statistical analysis was performed using one-way ANOVA, and differences were considered to be significant at *p* < 0.05. Analysis was carried out using the statistical software package SPSS, version 17.0 (SPSS 17.0 for Windows, SPSS Inc., Chicago, IL, USA). All studies were performed at least in triplicate and the data are presented as mean ± standard deviation.

## 4. Conclusions

In this study, the improved water solubility and anti-colon cancers activities of a curcumin analog, cyqualone (CL) was achieved by *N*-benzyl-*N*,*O*-succinyl chitosan (NBSCh) nanomicelles construction. The CL-loaded NBSCh nanomicelles were prepared by the dialysis method provided high loading capacity. The obtained nanomicelles were spherical. The release of CL from the obtained nanomicelles could be controlled by the pH environments. The blank NBSCh nanomicelles had low cytotoxicity on L929 and HT-29 cells. However, the CL-loaded NBSCh exhibited significantly higher anti-cancer activity against HT-29 colorectal cancer cells. The CL-loaded NBSCh nanomicelles were found stable for at least 120 days. Moreover, they were more effective by G2/M phase arresting cell growth and induced apoptosis earlier in HT-29 cells. Due to these aspects, the pH-sensitive CL-loaded NBSCh nanomicelles may have potential as a desirable candidate for targeted drug delivery to the colon and have anti-colon cancer activity. Nevertheless, for clinical application of the CL-loaded NBSCh nanomicelles, in vivo experiments are needed for further evaluation.

## Figures and Tables

**Table 1:** Elemental analysis of Chitosan, NBCh and NBSCh performed on CHN/O analyzer for DSB and DSS calculation.

Sample	%C	%H	%N	%O	C/N	DSB	DSS
Chitosan	49.9	7.57	7.01	35.52	7.12	-	-
NBCh	60.22	10.62	5.49	23.67	10.97	0.55	-
NBSCh	56.89	9.97	3.91	29.23	15.83	0.55	1.02

DSB: degree of benzyl substitution; DSS: degree of substitution with succinyl radical.

**Table 2:** Size, PDI values, Zeta-potential, encapsulation efficiency (%EE) and loading capacity (%LC) of NBSCh micelles using NBSCh 1 mg/mL.

Sample ID	Concentration of Loaded CL (mg/mL)	Size (nm)	PDI	Zeta Potential (mV)	%EE	%LC
Blank micelles	-	60.1 ± 0.7	0.096 ± 0.008	−29.3 ± 0.3	-	-
0.1-CL-micelles	0.1	59.0 ± 0.7	0.172 ± 0.018	−27.5 ± 1.4	44.4 ± 1.1	4.25 ± 1.1
0.3-CL-micelles	0.3	59.8 ± 0.6	0.186 ± 0.030	−30.3 ± 0.5	46.3 ± 1.0	12.20 ± 1.2
0.6-CL-micelles	0.6	62.4 ± 0.9	0.236 ± 0.022	−31.0 ± 0.6	45.1 ± 1.9	21.30 ± 1.5
1-CL-micelles	1	64.7 ± 0.7	0.272 ± 0.028	−30.3 ± 0.5	48.7 ± 1.3	32.75 ± 1.6

All the values are represented as mean ± SD., (*n* = 3).

**Table 3:** Cytotoxicity and selectivity of free cyqualone (CL) and CL-loaded NBSCh micelles against HT-29 human colon cancer cells and L929 mouse fibroblasts cell lines.

Sample	HT-29	L929	Selectivity
	IC_50_ (µg/mL)	IC_50_ (µg/mL)	(Fold)
Cyqualone (CL)	10.6 ± 1.14	10.2 ± 1.01 *	0.97 *
CL-NBSCh micelles	3.4 ± 0.82 *	24.3 ± 2.23	7.16

All the values are represented as mean ± std., * < 0.05 (*n* = 3).

## Data Availability

Not applicable.

## Supplementary Materials

The following supporting information can be downloaded at: https://www.mdpi.com/article/10.3390/molecules28062693/s1 Figure S1. FT-IR spectra (KBr) of (1) Chitosan (MW = 30,000), (2) *N*-benzyl chitosan (NBCh) and (3) *N*-benzyl-*N*,*O*-succinyl chitosan (NBSCh); Figure S2. 1H-NMR spectra (500 MHz) of Chitosan in D2O with acetic-D (1), *N*-benzyl chitosan (NBCh) in DMSO-d6 (2) and *N*-benzyl-*N*,*O*-succinyl chitosan (NBSCh) in DMSO-d6 (3); Figure S3. Graph for determination of CMC value of NBSCh micelle using Pyrene as a fluorescence probe.
