# Supplementary material for: Development of pH-Responsive N-benzyl-N-O-succinyl Chitosan Micelles Loaded with a Curcumin Analog (Cyqualone) for Treatment of Colon Cancer
Source: Molecules. 2023 Mar 16;28(6):2693. doi: 10.3390/molecules28062693 (PMC10057334; doi:10.3390/molecules28062693)
Supplement: Supplementary file 1 [file molecules-28-02693-s001.zip › molecules-2072906-supplementary.pdf]

molecules

Supporting information

Development of pH-Responsive *N*-benzyl-*N*-*O*-succinyl Chitosan Micelles Loaded with a Curcumin Analog (Cyqualone) for Treatment of Colon Cancer

Sasikarn Sripetthong[1,2], Chitchamai Ovatlarnporn [1,2,*] Fredrick Nwude Eze[1,2] and Warayuth Sajomsang[3]

[1] Department of Pharmaceutical Chemistry, Faculty of Pharmaceutical Sciences, Prince of Songkla University, Hat Yai, Songkhla 90112, Thailand
[2] Drug Delivery System Excellence Center, Faculty of Pharmaceutical Sciences, Prince of Songkla University, Hat Yai, Songkhla 90112, Thailand
[3] Nanodelivery System Laboratory, National Nanotechnology Center, National Science and Technology Development Agency, Phathum Thani 12120, Thailand
* Correspondence: chitchamai.o@psu.ac.th

Citation: Sripetthong, S.; Fredrick, N.E.; Sajomsang, W.; Ovatlarnporn, C.;Development of pH Responsive *N*-benzyl-*N*-*O*-succinyl Chitosan Micelles Loaded with a Curcumin Analog (Cyqualone) for Treatment of Colon Cancer. *Molecules* **2023**, *28*, 2693. https://doi.org/10.3390/molecules28062693

Academic Editor: Anne-Marie Caminade

Received: 17 November 2022
Revised: 19 February 2023
Accepted: 25 February 2023
Published: 17 March 2023

Copyright: © 2023 by the authors. Licensee MDPI, Basel, Switzerland. This article is an open access article distributed under the terms and conditions of the Creative Commons Attribution (CC BY) license (https://creativecommons.org/licenses/by/4.0/).

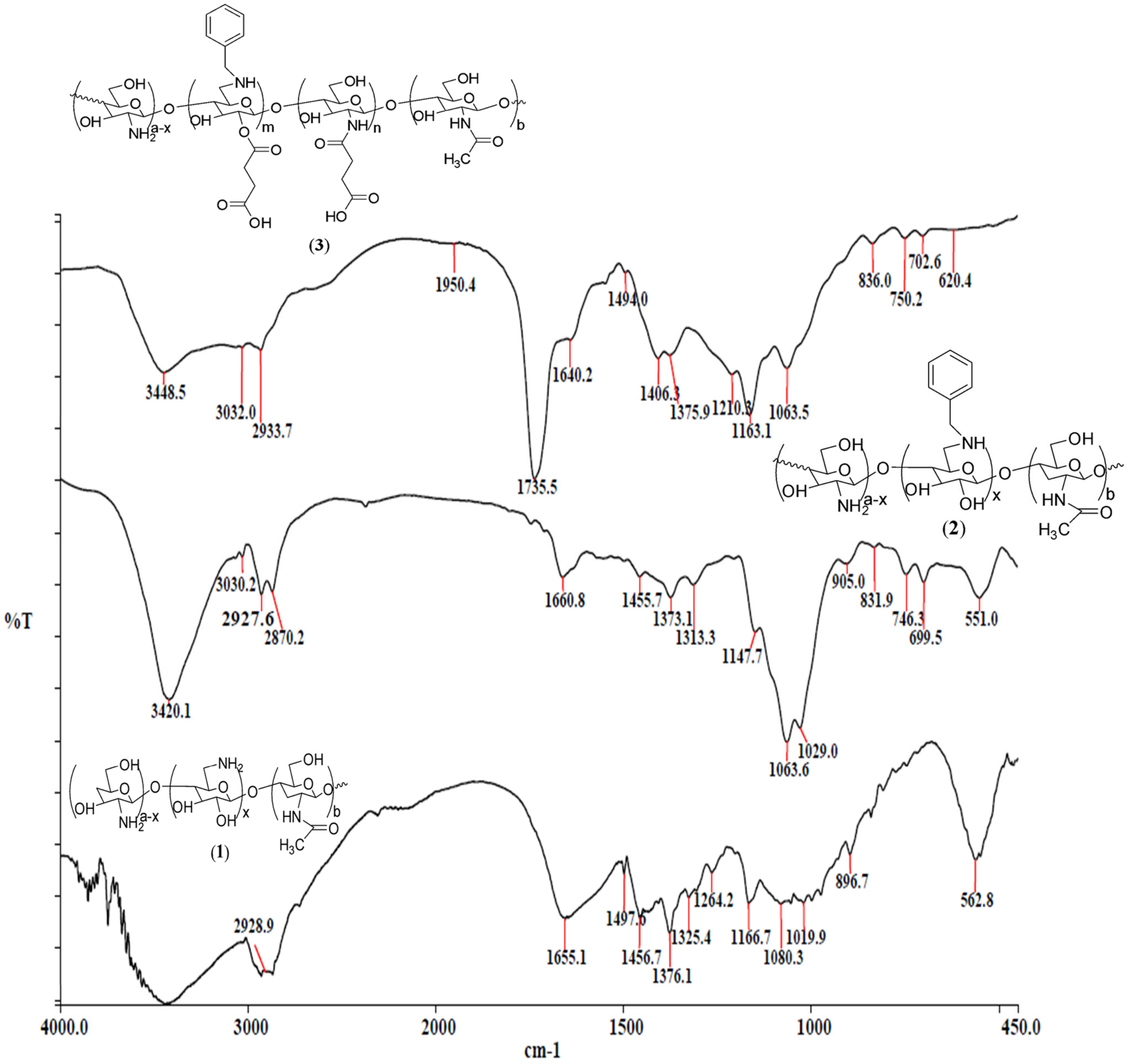

Figure S1. FT-IR spectra (KBr) of (1) Chitosan (MW = 30,000), (2) *N*-benzyl chitosan (NBCh) and (3) *N*-benzyl-*N,O*-succinyl chitosan (NBSCh)

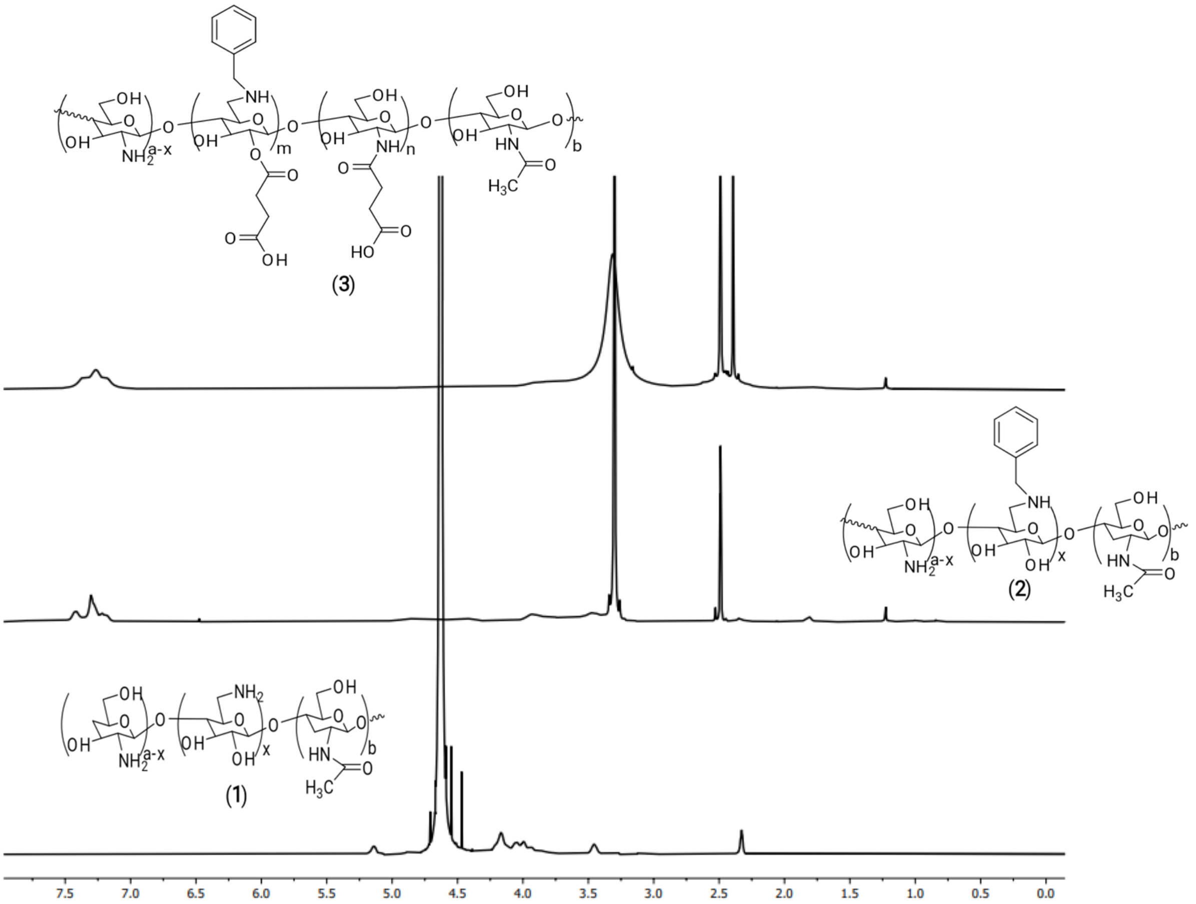

Figure S2. ^{1}H-NMR spectra (500 MHz) of Chitosan in D_2O with acetic-D (1), *N*-benzyl chitosan (NBCh) in DMSO-d_6 (2) and *N*-benzyl-*N*,*O*-succinyl chitosan (NBSCh) in DMSO-d_6 (3)

FT-IR spectrum of NBCh (Figure S1) displayed absorption bands at 1660.8 and 1455.7 cm^{-1} corresponding to C=C stretching of the aromatic group as well as absorption bands at 746.3 and 699.5 cm^{-1} attributed to C-H bending of the benzyl groups. Bands at 2870.2 and 2927.6 cm^{-1} are assigned to CH- stretching of alkyl groups in the benzyl groups and chitosan backbone. The broad band located at 3420.1 cm^{-1} corresponded to -OH stretching of the chitosan backbone. The broader band at 3448.5 cm^{-1} in the NBSCh spectrum emerged from -OH stretching of the chitosan backbone and the carboxylic group (-COOH) of substituted succinyl groups. Peaks at 3032.0 and 2933.7 cm^{-1} are assigned to -CH stretching of alkyl groups in the chitosan backbone, -CH_2- of benzyl groups and -CH_2-CH_2 of succinic groups. Peaks at 1735.5 cm^{-1} belonged to C=O stretching of the succinic acid moiety The peak at 1640.2 cm^{-1} belonged to -C=C- of benzyl groups, that at 1163.0 cm^{-1} belonged to -C-N- stretching, while that at 749.5 and 702.1 cm^{-1} are due to -CH bending.

The ^{1}H-NMR spectrum of NBCh (Figure S2) comprised of multiple peaks in the range of 7.20–7.50 ppm. These belonged to protons of the aromatic benzene ring of the substituted benzyl groups. The ^{1}H-NMR profile of NBSCh in DMSO-d_6 (Figure S3) was similar to the NBCh spectrum with only slightly differences. In addition to the peaks displayed by NBCh, a new peak was observed at 2.45 ppm in NBSCh. This peak was attributed to the -CH_2-CH_2- of the substituting succinic moieties.

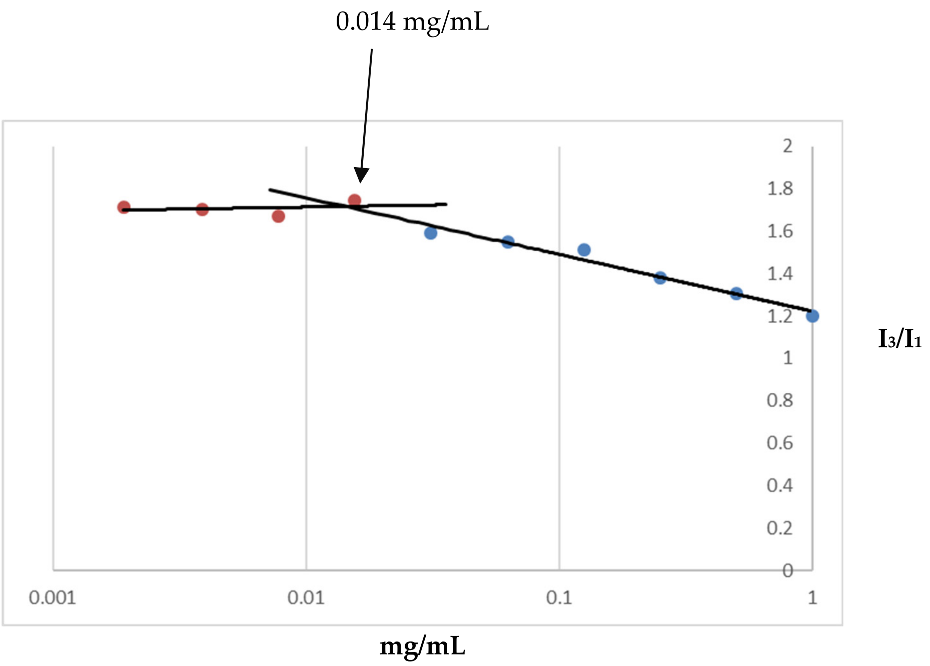

Figure S3. Graph for determination of CMC value of NBSCh micelle using Pyrene as a fluorescence probe.
